# Interventions for smoking cessation: An overview of Cochrane reviews

**DOI:** 10.18332/tid/195302

**Published:** 2024-11-28

**Authors:** Chun-li Lu, Jia-xuan Li, Qian-yun Wang, Rui-ting Wang, Xing-ru Pan, Xiao-ying Chen, Chao-jie Wang, Rui-lin Chen, Si-hong Yang, Zhi-hui Zhao, Jing-jing Jiang, Xue-han Liu, Jian-hua Wang, Xue Xue, Li-rong Liang, Nicola Robinson, Jian-ping Liu

**Affiliations:** 1 Centre for Evidence-Based Chinese Medicine, Beijing University of Chinese Medicine, Beijing, China; 2 Guangdong Provincial Research Center of Integration of Traditional Chinese Medicine and Western Medicine in Metabolic Diseases, Guangdong Pharmaceutical University, Guangzhou, China; 3 Key Laboratory of Glucolipid Metabolic Disorder, Ministry of Education, Guangzhou, China; 4 School of Clinical Traditional Chinese Medicine, Hubei University of Chinese Medicine, Wuhan, China; 5 Dongfang Hospital, Beijing University of Chinese Medicine, Beijing, China; 6 Cardiovascular Department Ward 3, The Second Affiliated Hospital of Shaanxi University of Chinese Medicine, Xianyang, China; 7 Dongzhimen Hospital, Beijing University of Chinese Medicine, Beijing, China; 8 Department of Integrative Oncology, China-Japan Friendship Hospital, Beijing, China; 9 Acupuncture and Moxibustion Massage College, Liaoning University of Traditional Chinese Medicine, Liaoning, China; 10 Department of Traditional Chinese Medicine, Xiangyang No.1 People's Hospital, Hubei University of Medicine, Hubei, China; 11 China Center for Evidence Based Traditional Chinese Medicine, China Academy of Chinese Medical Sciences, Beijing, China; 12 School of Nursing, Shanghai Jiao Tong University, Shanghai, China; 13 Graduate Institute of Interpretation and Translation, Shanghai International Studies University, Shanghai, China; 14 School of Traditional Chinese Medicine, Liaoning University of Traditional Chinese Medicine, Liaoning, China; 15 Department of Nephrology, Hubei Provincial Hospital of Traditional Chinese Medicine, Wuhan, China; 16 Department of Research on Tobacco Dependence Therapies, Beijing Institute of Respiratory Medicine and Beijing Chao-Yang Hospital, Capital Medical University, Beijing, China; 17 Institute of Health and Social Care, London South Bank University, London, United Kingdom

**Keywords:** overview, systematic review, smoking cessation, tobacco control, Cochrane review

## Abstract

**INTRODUCTION:**

Evidence of different smoking cessation interventions varies and has been assessed in many Cochrane reviews. We conducted an overview of these Cochrane reviews to summarize the effects of current interventions for smoking cessation.

**METHODS:**

Nine databases were searched from their inception to October 2024, with no restrictions on language. Two authors independently extracted data from the same studies simultaneously, double checking after extraction. A second round of examination was conducted on all the extracted contents by another author. We employed a measurement tool to assess systematic reviews (AMSTAR-2) to evaluate the methodological rigor of the included systematic reviews (SRs), synthesized the GRADE results as reported, and conducted a narrative synthesis. The research protocol was registered on PROSPERO (CRD42023388884).

**RESULTS:**

Seventy-one Cochrane reviews involving 3022 trials were included in this comprehensive analysis. The two predominant smoking cessation interventions were pharmacotherapy (24 SRs) and non-pharmacological therapy (31SRs). Overall, the methodological quality of all the reviews was good. Compared with placebo, the point effect size for each Cochrane review on relative risk (RR) regarding pharmacotherapies for prolonged abstinence rate ranged from 1.11 to 3.34, demonstrating high- or moderate-certainty evidence; whereas for non-pharmacological therapies, it varied from 0.79 to 25.38, but substantial heterogeneity was observed in most meta-analysis (I2>50%). Four studies investigating pharmacotherapies as interventions, adverse events were reported but no significant differences in outcomes were observed.

**CONCLUSIONS:**

Pharmacotherapy demonstrated some efficacy in promoting prolonged abstinence rate, while the effectiveness of different non-pharmacological interventions for smoking cessation varied widely, highlighting the need for further research on the integration of pharmacotherapy and non-pharmacological therapies.

## INTRODUCTION

Nicotine dependence is recognized as a chronic non-communicable disease^1^, associated with many other diseases with high morbidity and mortality^2^. It has been included in the International Classification of diseases (ICD-11) by the World Health Organization (Disease number 6C4A.2)^3^. The development of nicotine dependence is related to motivation, psychology, behavior and living environment of smokers^1^. Therefore, interventions for smoking cessation primarily focus on addressing nicotine dependence and modifying behavioral habits. Current approaches for smoking cessation can be categorized into pharmacological therapy and non-pharmacological therapy.

Pharmacotherapy encompasses nicotine replacement therapy (NRT) and non-NRT. Nicotine replacement products such as nicotine patches, nicotine chewing gum, and nicotine buccal tablets can alleviate nicotine withdrawal symptoms by delivering nicotine to the body in a compensatory manner for short-term stimulation. NRT is recommended as a first-line treatment for smoking cessation in many national guidelines^4^. Non-NRT are defined as drugs without nicotine but can be used as smoking cessation treatments. Bupropion and varenicline are the primary non-NRT treatments with distinct mechanisms of action^5,6^. As a non-tricyclic antidepressant, bupropion can mitigate anxiety, depression, and other withdrawal symptoms by inhibiting nicotinic acetylcholinergic receptors. Thus, it is advised that smokers initiate its use one week prior to quitting. Varenicline acts as a partial agonist of the α4β2 nicotinic acetylcholine receptor in the brain similar to nicotine, so it can reduce nicotine craving and withdrawal symptoms associated with nicotine dependence^5^. Previous studies have shown varenicline’s superiority over placebo, while also highlighting common adverse effects such as nausea, dry mouth, gastrointestinal discomfort, and associated suicidal tendency^7,8^. Despite its efficacy, attention must be given to varenicline’s post-withdrawal adverse effects.

Non-pharmacological interventions include brief counseling-based intervention models and various behavioral intervention models implemented in other different ways. The 5As (Ask, Advise, Assess, Assist, and Arrange) and 5Rs (Relevance, Risk, Rewards, Roadblocks, and Repetition) models serve as the predominant theoretical framework for smoking cessation^5^. These fundamental steps of 5As and 5Rs models underpin the majority of non-pharmacological interventions. Previous studies have demonstrated that any form of behavioral intervention can effectively enhance motivation and facilitate smoking cessation^9^. Moreover, although there is a lack of conclusive evidence to support this claim, combining behavioral interventions with pharmacotherapy may potentially improve quit rates^10^.

The interventions recommended by the World Health Organization (WHO) encompass psychological counseling, behavioral interventions, and western medicine treatment. However, there is a lack of recommendations for different populations, single or integrated intervention methods, and outcome efficacy indicators^11^. Upon comparing clinical practice guidelines from various countries around the world^12,13^, it was observed that most guidelines advocate counseling or behavioral support, while only a few guidelines recommended smoking cessation medications without sufficient evidence-based support^4,5^.

Cochrane has consistently advocated for the utilization of high-quality evidence to inform clinical decision-making. Reviews conducted under its rigorous methodological guidance and stringent requirements, are universally acknowledged as the epitome of evidence-based information. This study aimed to provide an overview of Cochrane reviews, summarizing the most current and robust evidence on smoking cessation interventions while also evaluating the quality of evidence.

## METHODS

This study followed the methodological process in the ‘Overview of Systematic Reviews’ of the Cochrane Handbook^14^ and is reported in accordance with the PRISMA 2020^15^. The research protocol was prior to register on PROSPERO (CRD42023388884).

### Eligibility criteria

*Study design*

Cochrane reviews or overviews published in full text were included without any restrictions on the type of original studies included in the systematic reviews (randomized controlled trial, non-randomized controlled trial, qualitative research, etc.). In cases where multiple versions of the same Cochrane review existed due to updates, only the most recent version was considered. Given Cochrane’s publication policy, authors have the flexibility to incorporate original data and submit it for publication in other journals. To ensure comprehensive information coverage, studies meeting the inclusion criteria from other journals were also incorporated. Additionally, if a full-text article had been withdrawn, its citation information was still retained.

*Participants*

People who had smoked or had nicotine dependence, regardless of tobacco used types, were included. People exposed to secondhand or thirdhand smoke (i.e. passive smoking) were excluded.

*Interventions*

There were no restrictions on interventions, including NRT (nicotine patch, nicotine chewing gum, etc.), non-NRT (bupropion, varenicline), non-pharmacological therapy (counselling, behavioral therapy, psychotherapy, etc.), policies on tobacco cessation, complementary and alternative treatment such as traditional Chinese medicine, and other therapies. Interventions could be employed either independently or in conjunction with one another.

*Comparisons*

There was no limitation on the type of control settings.

*Outcomes*

The included studies contained abstinence rate as one of the outcomes (including continuous abstinence, prolonged abstinence, prolonged abstinence with lapses, point-prevalence abstinence, repeated point-prevalence abstinence, etc.)^16^. Meanwhile, other outcomes related to tobacco use, such as the evaluation of nicotine withdrawal symptoms, relapse rate, smoking index, self-efficacy assessment, and safety outcomes were allowed.

### Search strategy and databases

To ensure comprehensiveness, we searched nine databases (Cochrane Database of Systematic Reviews (CDSR), PubMed, Embase, Web of Science, SinoMed, the Chinese National Knowledge Infrastructure Databases (CNKI), the China Science and Technology Journal Database (VIP), Wanfang Data Knowledge Service Platform, and EPISTEMONIKOS from their inception to 13th October 2024.

The search strategy was specified according to the standard of Cochrane search group. Key words were: ‘smoking cessation’, ‘tobacco cessation’, ‘stop smoking’, ‘quit smoking’, ‘preventing smoking’, ‘nicotine dependence’, ‘smoking reduction’, ‘cigarette smoking cessation’, ‘systematic review’, ‘meta-analysis’, ‘overview’, ‘rapid review’, ‘umbrella review’ and ‘mapping review’. There was no restriction on the language of publication. The search strategy details are presented in Supplementary file Table 1.

### Study selection

All the original citations were exported to NoteExpress software (version 3.5.0.9054). for management and organization purposes. Following the removal of duplicates, two authors independently screened titles and abstracts to identify studies that potentially met the pre-defined inclusion criteria mentioned above. Subsequently, the full-texts were individually assessed by two authors to determine their eligibility for inclusion in this study. Any disagreement was resolved through discussion with a third author. In cases where Cochrane reviews had been withdrawn, we retained only the citation information while providing a concise summary of the reasons for their withdrawal.

### Data extraction

Data were extracted by using a predesigned form in WPS (version: Spring 2022 update, 11636). The pre-extraction process was conducted by two authors who had received training on data extraction and then began to extract data independently in duplication. Any disagreement in the process was resolved through discussion with a third author. To ensure accuracy, the data were cross-checked by the third author. Extracted information included:

1. Basic information, such as publication year, author information, number of included studies, study type of included studies, funding sources, retrieval time, settings, etc.
2. Clinical information on participants (sample size, population characteristics, disease status, average daily number of smokes, etc.), intervention (types, implementation method, mechanism of action, duration, follow-up duration, etc.), and outcomes.
3. Information on effect sizes: meta-analysis, subgroup analysis, summary results of abstinence rate, nicotine withdrawal symptoms, relapse rate, etc.

### Methodology assessment

The methodological quality of the included reviews was assessed using the A measurement tool to assess systematic reviews (AMSTAR-2)^17^. Two authors independently conducted the assessment, which was then cross-checked by a third author. Any discrepancies were resolved through discussion with a third author. Each item was evaluated as ‘Yes’, ‘No’, ‘Partial Yes’ or ‘No meta-analysis’ (for details of the 16 items see Supplementary file Table 2). The overall quality of the reviews was evaluated as ‘High’, ‘Moderate’, ‘Low’, ‘Critically low’ based on the key items. Considering the trust in Cochrane reviews’ quality, we did not re-evaluate the certainty of evidence from original meta-analysis but rather summarized the results of GRADE.

### Data analysis

The qualitative data were subjected to content analysis for combination and classification. The basic characteristics of the studies were combined by counting. Given the considerable clinical heterogeneity of interventions included in the reviews, we employed a summary of evidence approach to analyze the quantitative data. The data included abstinence rate, nicotine withdrawal symptoms and relapse rate. For dichotomous outcomes, relative risk (RR) or odds ratio (OR) with 95% confidence intervals (CI) were utilized as presentation measures, while mean difference (MD) with 95% CI was used for continuous outcomes.

## RESULTS

### Study selection

**Figure 1:**
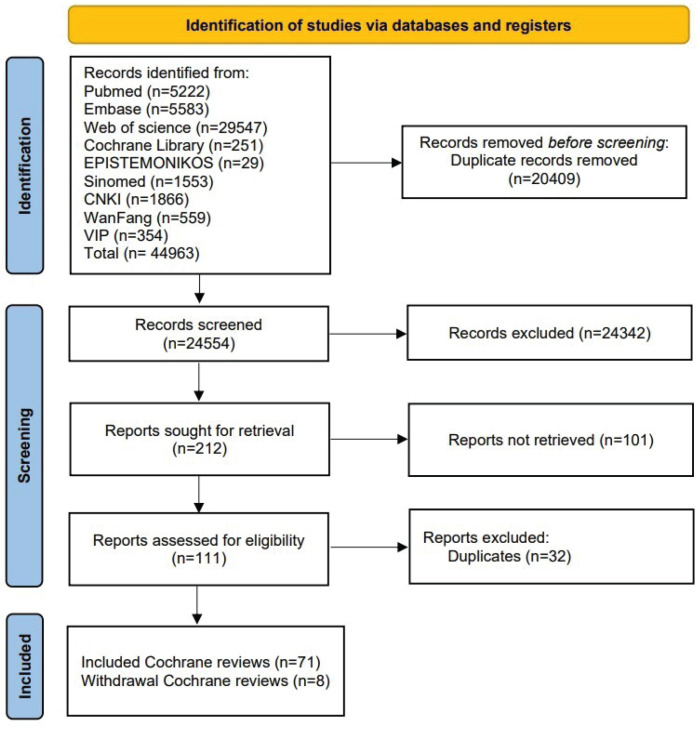
Flow diagram of the overview of Cochrane reviews

A total of 44963 records were identified and after removing duplicates, 24554 publications remained. Following the screening of titles, abstracts, and full-text, a final selection of 71 studies^18-87^ was included (Figure 1). Additionally, we retained the citation information for eight withdrawn systematic reviews and recorded the reasons for their withdrawal (Supplementary file Table 3).

### Basic information of the included studies

**Figure 2:**
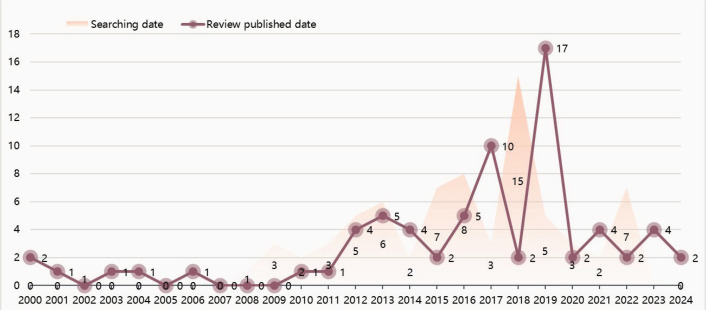
The publication time and corresponding retrieval time of the 71 Cochrane reviews

The first review was published in 2000, and since then there has been a gradual increase in the number of publications, with a sharp decline observed by 2020. The distribution of the search time and publication time for the reviews were found to be largely consistent (Figure 2). Government agencies, universities, and other foundations were identified as the primary sources of funding for included reviews. Notably, the University of Oxford received the largest share of research funding from the UK National Institute for Health Research (45/71; 63.38%).

The institution with the highest number of first authors was Oxford University, and the majority of the studies did not involve Chinese authors (69/71; 97.18%). The United States and Australia were the countries or regions that received the most funding and conducted extensive research on smoking cessation (Supplementary file Figure 1).

Amongst the 71 Cochrane reviews, a total of 3022 trials were included, with a predominant focus on randomized controlled trials (2744/3022; 90.80%). Of the remaining studies, 75 were observational studies, eight were non-RCTs, eight were quasi-experimental studies, and 246 were ongoing studies. Clinics in medical institutions served as the most common setting for these interventions (36/71; 50.70%), followed by communities (26/71; 36.62%) (Table 1). Seven SRs concluded that the smoking cessation interventions were ‘effective’, and forty-nine SRs reported them to be ‘potentially effective’. In contrast, five reviews deemed them ‘potential ineffective’, seven concluded they were ‘ineffective’, but three studies did not include any of the original studies.

**Table 1:** Basic information of the 71 included Cochrane reviews

*Report information*	*Trials (N=3022) n (%)*
Type of included studies*	
RCTs	2744 (90.80)
Observational studies	75 (2.50)
Cohort studies	15 (0.50)
Non-RCTs	9 (0.30)
No. of quasi-experimental studies	8 (0.26)
Cross-sectional studies	1 (0.03)
Ongoing studies	259 (8.57)
Publication language of original studies included in SRs*	
English	36 (1.19)
Chinese	3 (0.10)
French	5 (0.17)
Italian	1 (0.03)
Unclear	2977 (98.51)
***Settings***	***Reviews (N=71) n (%)***
**Medical institutions**	
Clinic	35 (49.30)
Hospital	17 (23.94)
Healthcare setting	6 (8.45)
Pharmacy	3 (4.23)
Substance abuse treatment sites	1 (1.41)
Automated healthcare records center	1 (1.41)
**Research institutions**	
Research center/medical center	10 (14.08)
International center	1 (1.41)
Clinical laboratory	1 (1.41)
**Social institutions**	
Community	25 (35.21)
University	9 (12.68)
Homeless shelter	2 (2.82)
Government	1 (1.41)
Military	1 (1.41)
Air force	1 (1.41)
**Other places**	
High school/secondary school	5 (7.04)
Home	5 (7.04)
Worksites	8 (11.27)
Online	2 (2.82)
Anywhere	2 (2.82)
Village	1 (1.41)

* Type of included studies and publication language were derived from the original studies in the systematic review. RCT: randomized controlled trial. SR: systematic review.

### Clinical characteristics of included studies

The sample size of participants in SRs ranged from 128 to 33000. Among the included studies, 19 studies (24.76%) did not provide detailed information about participants characteristics, while the remaining studies reported various aspects such as age (51/71; 71.83%), occupation (6/71; 8.45%), pre-existing conditions (16/71; 22.54%), daily tobacco consumption (24/71; 33.80%), and pregnancy status (18/71; 25.35%) of the participants studied (Table 2).

**Table 2:** Characteristics of participants of the 71 included Cochrane reviews

*Participant information*	*n (%)*
**Characteristics of participants**	
Unlimited	19 (26.76)
Adult	20 (28.17)
Participants aged <15 years	1 (1.41)
Participants aged 18–65 years	1 (1.41)
Participants aged <20 years	1 (1.41)
Attempt to quit smoking	7 (9.86)
No attempt to quit smoking	1 (1.41)
Pregnant	2 (2.82)
Non-pregnant	7 (9.86)
**Social status**	
Adult with stable work	1 (1.41)
Medical practitioner	1 (1.41)
Community healthcare worker	1 (1.41)
Community pharmacy worker	1 (1.41)
Adult experiencing homelessness	1 (1.41)
Healthcare patients and workers	1 (1.41)
**Pre-existing conditions**	
Unlimited (not reported)	54 (76.06)
Mental health problems	8 (11.27)
People with substance abuse problems	7 (9.86)
Chronic obstructive pulmonary disease	4 (5.63)
Cardiovascular disease or at high risk of developing heart disease	4 (5.63)
HIV-positive and AIDS	3 (4.23)
Cancer	3 (4.23)
Diabetes	3 (4.23)
Respiratory disease (not specific)	2 (2.82)
Tuberculosis	2 (2.82)
Chest disease	2 (2.82)
Heart surgery	2 (2.82)
Surgical patients	2 (2.82)
Asthma	1 (1.41)
Chronic inflammatory arthropathy	1 (1.41)
Varenicline in pregnancy	1 (1.41)
**Cigarettes per day**	
Not reported	46 (64.79)
<11	10 (14.08)
11–20	4 (5.63)
21–30	5 (7.04)
>30	5 (7.04)

In terms of interventions, the included SRs involved various approaches such as pharmacotherapy (24 SRs), non-pharmacological therapy (31 SRs), mass media (2 SRs), traditional Chinese medicine (TCM) therapy (1 SR), policy (5 SRs), other support (6 SRs) and combinations of different therapies (12 SRs). Notably, NRT (10/71; 14.08%), varenicline (8/71; 11.27%) and bupropion (8/71; 11.27%) received significant attention among pharmacotherapies. Smoking cessation counseling emerged as the most extensively studied non-pharmacological intervention with seven studies identified out of a total of seventy-one reviews analyzed in this stud (7/71; 9.86%). The most common combination therapies were ‘drug plus behavioral interventions’ and ‘drug plus counseling’, each accounting for three out of seventy-one reviews (3/71; 4.23%). The duration of these interventions varied from one week to one year. In addition, an innovative intervention involving altering tobacco packaging to demonstrate the negative effects of smoking has been used for 18 years through public information dissemination^22,26^. Supplementary file Table 4 provides a summary of potential mechanisms to enhance the motivation underlying different interventions, including improving the efficacy of smoking cessation, reducing the relapse rate, developing new habits, and alleviating nicotine withdrawal symptoms. However, there is a lack of appropriate measures to evaluate the availability and effectiveness of smoke-free environments.

Among the primary outcomes, continuous abstinence rate (36/71; 50.70%) and point-prevalence abstinence (22/71; 30.99%) were the most common. The secondary outcome indicator that occurred most frequently was participant adherence to the intervention (14/71; 19.72%). Safety evaluation primarily focused on western drugs, with adverse events reported in 23 out of 71 reviews, (32.39%) (Table 3).

**Table 3:** Summary of outcomes of the 71 included Cochrane reviews

*Outcome*	*n (%)*
**Primary outcomes**	
Continuous abstinence	36 (50.70)
Point prevalence tobacco abstinence	22 (30.99)
Smoking cessation	19 (26.76)
Smoking abstinence in late pregnancy	1 (1.41)
Changes in smoking prevalence	1 (1.41)
Cigarette sale	1 (1.41)
Changes in smoking behavior	1 (1.41)
Quality of life	1 (1.41)
Lung function	1 (1.41)
Disease activity score	1 (1.41)
**Secondary outcomes**	
Adherence to intervention	14 (19.72)
Quit attempts	7 (9.86)
Mental health and well-being	6 (8.45)
Smoking prevalence	6 (8.45)
Fitness (including mental state, oral health, survival, etc.)	6 (8.45)
Tobacco consumption	5 (7.04)
Withdrawal, reinforcing or hedonistic effects of smoking	4 (5.63)
Biochemical indicators	3 (4.23)
Changes in smoking cessation support actions	3 (4.23)
Abstinence from alcohol and other drugs	2 (2.82)
BMI and body weight	2 (2.82)
Abstinence from smoking after childbirth	1 (1.41)
Satisfaction of treatment	1 (1.41)
The effectiveness of therapeutic alliance	1 (1.41)
**Safety outcomes**	
Nausea, dizziness, fatigue	6 (8.45)
Number of deaths	4 (5.63)
Adverse event of emotion	3 (4.23)
Incidence of respiratory disease	3 (4.23)
Incidence of cardiovascular disease	2 (2.82)
Incidence of gastrointestinal disturbances	2 (2.82)
Incidence of other diseases	1 (1.41)
Incidence of liver disease	1 (1.41)
Adverse event in pregnancy	1 (1.41)
Disability (function)	1 (1.41)

### Methodological quality of included SRs

**Figure 3:**
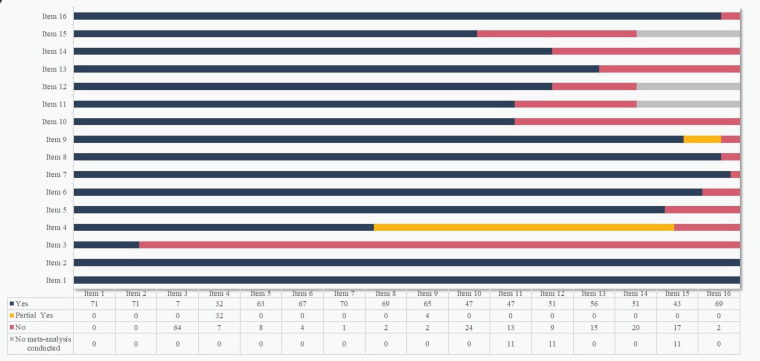
Assessment the included 71 Cochrane reviews by AMSTAR-2

The methodological quality of each study was assessed by AMSTAR-2 (Supplementary file Table 2). All the studies contained the following elements: participants (P), interventions (I), comparisons (C) and outcomes (O), and reported research protocols. However, a majority of the studies (63/71; 88.73%) lacked explanation of the selection of the study designs for inclusion (Item 3), while almost half of them (32/71; 45.07%) were considered deficient in comprehensive literature search strategy (Item 4) due to limited database searches or lack of consultation experts in the relevant fields. Overall, the quality was deemed good, with 52 studies classified as moderate-quality, 11 as high-quality, and 8 as low-quality (Figure 3).

### GRADE summary of interventions

A total of 45 SRs comprising 169 meta-analyses, reported GRADE assessment. Evidence from 23 meta-analyses was classified as high quality, while 55 were rated as moderate quality. Additionally, 63 meta-analyses were rated as low quality, and 28 meta-analyses were rated as very low quality (detailed information on each meta-analysis in Supplementary file Table 5).

### Effects of interventions

**Figure 4:**
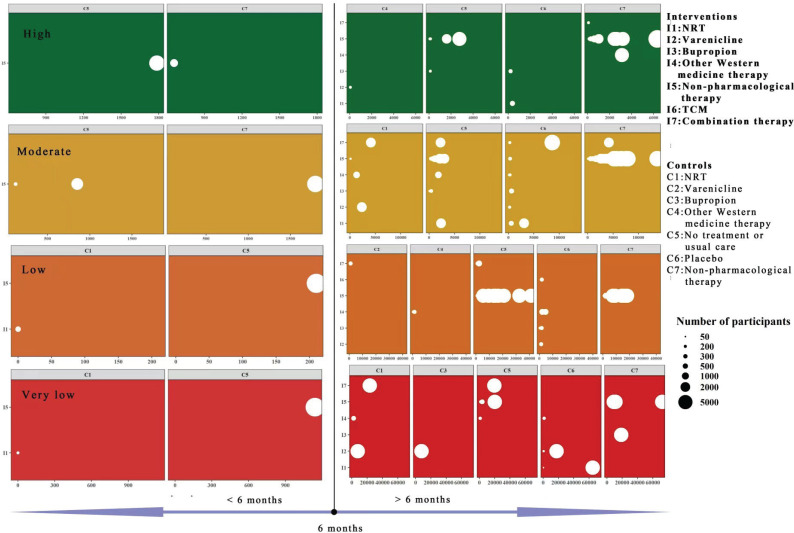
Bubble chart of interventions for smoking cessation

Among the 71 SRs, only abstinence rate was reported, with no studies reporting effect size results for nicotine withdrawal symptoms or relapse rate. The abstinence rate included various measures such as continuous abstinence rate, point-prevalence abstinence rate, repeated point-prevalence abstinence rate, and abstinence rate without clear criteria. Supplementary file Table 5 provides detailed information on each outcome included in the meta-analysis. The measurement time points ranged from one month to 48 months, with six months being the most commonly used time point to assess smoking cessation treatment efficacy. Figure 4 presents a bubble chart illustrating the variation in sample size, intervention and comparison groups, and follow-up duration (more than or less than 6 months), based on different levels of GRADE certainty.

*Prolonged abstinence*

Among the 21 SRs included, the prolonged abstinence rate was reported with varying RR, depending on different interventions and measurement time points. For non-pharmacological therapies, RR ranged from 0.79 to 25.38. However, most studies had confidence intervals that crossed the validity line and exhibited high heterogeneity (I2>50%). Therefore, further investigations are warranted to investigate the potential effectiveness of non-pharmacological therapies in achieving prolonged abstinence rates. On the other hand, pharmacotherapies demonstrated RR ranging from 1.11 to 3.34, with the majority of studies being classified as ‘moderate’ or ‘high’ quality, which suggests that pharmacotherapies may have potential advantage in improving the prolonged abstinence rate. Additionally, when combined with non-pharmacological therapies, pharmacotherapies showed RR ranging from 0.77 to 2.76, and several studies^38,47,73,78^ were rated as ‘high’ or ‘moderate’ quality for these combination interventions.

*Repeated point-prevalence abstinence*

Seven SRs reported repeated point-prevalence abstinence as an assessment measure to evaluate the effectiveness of smoking cessation interventions. The majority of studies provided evidence of low or very low quality, and the reported interventions did not demonstrate a significant impact on cessation rates.

*Point-prevalence abstinence*

The effect of interventions on the point-prevalence abstinence was analyzed by 14 SRs. Non-pharmacological therapies including behavioral interventions, counselling, or hypnotherapy, demonstrated a potential advantage in increasing the point-prevalence abstinence rate with RR ranging from 1.25 to 19.0. However, the conclusions are weakened by low quality evidence and uncertainty remains regarding the short-term efficacy of non-pharmacological therapies.

*Abstinence where the time point and method of measurement were not clear*

The abstinence was assessed by four SRs. However, they did not provide explicit time points or details regarding the measurement method.

*Adverse events*

Adverse events were reported in four studies, all of which investigated pharmacotherapies as interventions. RR for adverse events varied based on the type of interventions, dosage, duration of use, and the control measures employed.

## DISCUSSION

As widely acknowledged, Cochrane reviews are globally recognized as the gold standard for health guidelines^88^. The rigorous methodology and the regular updates ensure the provision of cutting-edge insights. Notably, Cochrane reviews exert a significant impact on specialized areas, with over 30 studies included in our analysis being updated more than three times. Such frequent updates pose a challenge to any systematic review endeavor. Regrettably, the forthcoming disbandment of the Cochrane Tobacco Addiction Group in 2023 will undoubtedly constitute a substantial loss to smoking cessation guidelines across various domains. In order to obtain a comprehensive understanding of the current landscape of smoking cessation interventions, our research meticulously compiled all existing Cochrane reviews involving 3022 original trials.

During the literature screening, we identified two reviews that were withdrawn due to potential conflicts of interest related to commercial affiliations. Given that funding sources can impact the credibility of study conclusions, it is crucial to thoroughly assess potential conflicts of interest (details in Supplementary file Table 3). Notably, the University of Oxford received substantial financial support funding (Supplementary file Figure 1), which could be attributed to the UK’s pioneering role in tobacco control campaigns. Consequently, British authors may have been well-position to conduct a more extensive investigation on smoking cessation. However, it is worth mentioning that all Cochrane reviews were conducted by the Cochrane Tobacco Addiction Group, whose editorial members are all from the Nuffield Department of Primary Health Sciences at the University of Oxford. This concentration within a single author team might have led to incomplete searches, and subsequently influenced AMSTRA-2 assessment results regarding comprehensive search strategies (Item 4). To enhance clinical practice utilization of evidence synthesis, it is imperative to prioritize comprehensive and transparent reporting in living review, rapid review, living guideline, and rapid guidelines.

In terms of current smoking cessation clinical trials design, the randomized controlled trial remains the optimal approach for verifying intervention effectiveness. Regarding the summary of clinical trials settings in the reviews, outpatient clinics were found to be the most frequently utilized setting, offering accessible brief smoking cessation interventions due to their unique location. Future clinical trials should prioritize effectively promoting the role of the outpatient clinics in smoking cessation. The reviews indicated that most studies did not impose restrictions on participants’ disease status or age, suggesting that excessive limitations may hinder promotion and application in clinical practice. Patient-centered intervention design is crucial for successful smoking cessation, with patient adherence playing a significant role in management. In the era of digital healthcare, traditional clinical trials alone are insufficient for controlling smoking cessation; instead, complex interventions incorporating long-term follow-up and innovative study designs that integrate digital healthcare are necessary to introduce implementation science methodology and enhance intervention fidelity^89,90^. Furthermore, future clinical trials should address multi-morbidity smokers such as those with chronic obstructive pulmonary disease and cardiovascular/cerebrovascular diseases, who have an urgent need for effective treatment to quit smoking.

Topic interventions for smoking cessation vary annually, while the methods of cessation have become more standardized, encompassing medication to counseling and behavioral interventions. The introduction of the 5As and 5Rs approaches, has aimed to standardize the behavioral and counseling strategies for smoking cessation. Additionally, combination treatments involving both behavioral and pharmacological interventions have demonstrated superior efficacy based on current reviews. Pharmacotherapy including varenicline, bupropion, and NRT generally exhibits more pronounced efficacy compared to non-pharmacological therapy. Varenicline is particularly recommended as the most effective treatment in the clinical guidelines. However, despite their proven efficacy, concerns regarding safety have led some companies to discontinue production of these drugs. Moreover, uncertainty remains regarding the effectiveness of different non-pharmacological interventions. Future studies on smoking cessation intervention should explore a complex model combining drug therapy with behavior modification rather than relying solely on monotherapy to enhance abstinence rates. Furthermore, Figure 4 highlights insufficient demonstration of high certainty in clinical trials. Thus, further trials must prioritize high-quality design along with rigorous implementation and transparent reporting to ensure robust evidence.

Regarding the outcomes, unanimous acceptance among authors was observed only for the abstinence rate as the primary outcome measure. However, existing studies and reviews have failed to establish a standardized and unified definition of the abstinence rate. Future research should focus on establishing consistent calculation methods and measurement time points for assessing abstinence rates. Additionally, attention should be given to understanding nicotine withdrawal symptoms during cessation and relapse after cessation, as these factors significantly impact patient adherence.

### Strengths and limitations

Previous evidence-based evaluation studies have traditionally focused on a single type of smoking cessation intervention or participant group, limiting the scope of their findings^91^. Cochrane reviews are globally recognized as the gold standard for evidence-based research. For over two decades, the Cochrane Tobacco Addiction Group has played a crucial role in evaluating interventions for treating and preventing tobacco addiction, providing valuable support for policy development and clinical practice guidelines worldwide^92^. However, regrettably, the group was disbanded in March 2023. Therefore, this presents an opportune moment to consolidate all current Cochrane reviews. Through this comprehensive overview, we systematically examined the existing evidence from relevant Cochrane reviews to provide a thorough summary of efficacy and safety regarding smoking cessation interventions. Our aim is to present the current trends in effective smoking cessation treatment and offer implications for further clinical trials, guideline development, and policy-making.

Limitations also need to be addressed in our overview. One limitation of this study is the exclusive analysis of Cochrane reviews, which are globally recognized as high-quality evidence. However, due to the selection planning and long publication cycle time of the Cochrane Tobacco Addiction Group^93^, our study may not comprehensively cover all types of smoking cessation interventions or capture the latest state of clinical research, such as the digital technology combined with behavioral theory, complex interventions involving more than three treatment types, and multi-morbidity patients. Digital health solutions can aid in managing patients through mobile applications and wearable electronic devices to ensure high adherence and intervention fidelity. It should be noted that single interventions alone cannot guarantee effectiveness for smoking cessation. However, complex interventions with multistage arrangements have shown better efficacy compared to single interventions. Nevertheless, assessing effect size through meta-analysis was challenging due to significant heterogeneity among complex interventions across Cochrane reviews and clinical trials. Considering the good quality of Cochrane reviews, we did not analyze the original clinical studies included in the SRs, but focused on summarizing intervention data from SRs using the existing meta-analysis with GRADE, while also analyzing potential mechanisms underlying different interventions within each review (details provided in Supplementary file Table 4). Consequently, there may be overlap between original clinical studies included in different systematic reviews, which is another limitation of this overview. The future may witness the implementation of meta-analysis to ascertain the effectiveness of individual interventions or their combined application through amalgamating systematic reviews and primary studies.

## CONCLUSIONS

The Cochrane reviews serve as the primary source of high-quality evidence for guidelines, and a comprehensive overview of the current state and the efficacy of smoking cessation interventions. Both pharmacotherapy and non-pharmacological therapy are commonly employed interventions, with pharmacotherapies demonstrating superior effectiveness over placebo in achieving prolonged abstinence rates. However, the effectiveness of various non-pharmacological interventions for smoking cessation varies significantly, necessitating further research on combined therapies. Moreover, there is currently insufficient strong evidence to establish the association between pharmacological treatments and adverse events. Therefore, additional safety data are required for comprehensive evaluation.

## Supplementary Material



## Data Availability

All data used in this study are provided in the article and in the Supplementary file.
