# Supplementary material for: Interventions for smoking cessation: An overview of Cochrane reviews
Source: Tob Induc Dis. 2024 Nov 28;22:10.18332/tid/195302. doi: 10.18332/tid/195302 (PMC11603414; doi:10.18332/tid/195302)
Supplement: Supplementary file 1 [file TID-22-182-s1.pdf]

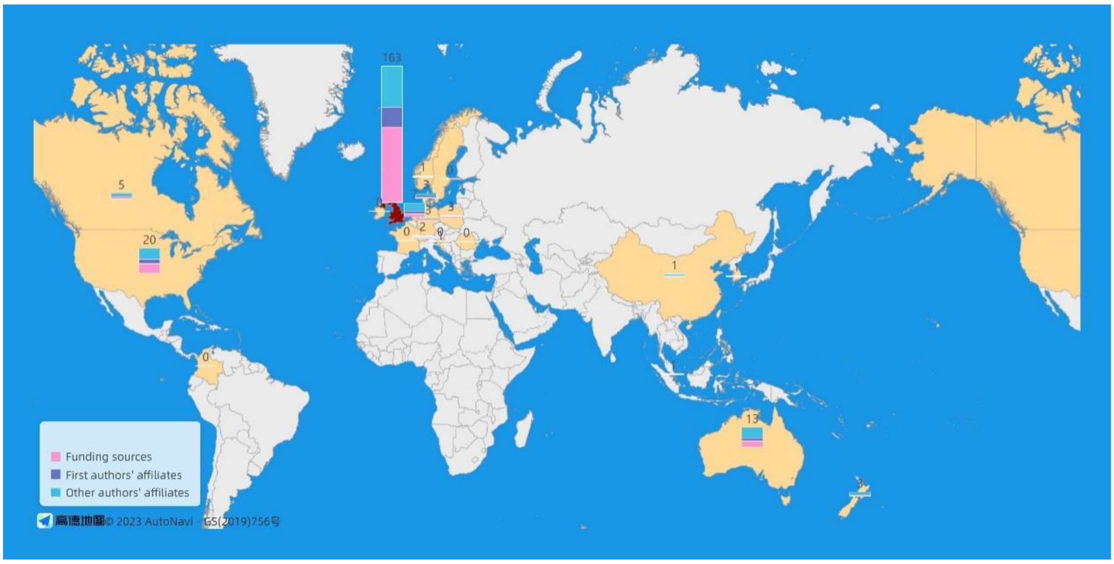

Supplementary material Figure 1 The country or region distribution of the funding sources and author units of the 71 included Cochrane reviews

Supplementary material Table 1 Search strategy (in Cochrane Library)

#1	SMOKING*:KW
#2	TOBACCO-USE-DISORDER*:KW
#3	TOBACCO-SMOKELESS*:KW
#4	TOBACCO-SMOKE-POLLUTION*:KW
#5	TOBACCO-USE-CESSATION*:KW
#6	NICOTINE*:KW
#7	(SMOKING and CESSATION):TI,AB,KW
#8	ANTISMOK*:TI,AB,KW
#9	QUIT*:TI
#10	SMOK*:TI
#11	CIGAR*:TI
#12	TOBACCO:TI
#13	NICOTINE:TI
#14	#1 or #2 or #3 or #4 or #5 or #6 or #7 or #8 or #9 or #10 or #11 or #12 or #13
#15	Filter-review
#16	#14 and #15

Other English databases：

#1	Smoking (MeSH Terms)
#2	Tobacco (MeSH Terms)
#3	#1 OR#2
#4	Smoking cessation (Title/Abstract)
#5	Cigarette smoking cessation (Title/Abstract)
#6	Tobacco cessation (Title/Abstract)
#7	Tobacco smoking cessation (Title/Abstract)
#8	Quit smoking (Title/Abstract)
#9	Stop smoking (Title/Abstract)
#10	Preventing smoking (Title/Abstract)
#11	Tobacco (Title/Abstract)
#12	Nicotine (Title/Abstract)
#13	Nicotine withdrawal (Title/Abstract)
#14	Nicotine dependence (Title/Abstract)
#15	Tobacco withdrawal syndrome (Title/Abstract)
#16	Passive smoking (Title/Abstract)
#17	Smoking habit (Title/Abstract)
#18	Cigarette smoking (Title/Abstract)
#19	Smokeless tobacco (Title/Abstract)
#20	Smoking reduction (Title/Abstract)
#21	Smoking prevention (Title/Abstract)
#22	Tobacco-Use-Cessation (Title/Abstract)
#23	Vaping (Title/Abstract)
#24	Electronic Nicotine Delivery Systems (Title/Abstract)
#25	Electronic cigarette (Title/Abstract)
#26	Pipe smoking (Title/Abstract)
#27	Tobacco Products (Title/Abstract)
#28	#4 OR #5 OR #6 OR #7 OR #8 OR #9 OR #10 OR #11 OR #12 OR #13 OR #14 OR #15 OR #16 OR #17 OR #18 OR #19 OR #20 OR #21 OR #22 OR #23 OR #24 OR #25 OR #26 OR #27
#29	Systematic review (Title/Abstract)

#30	Meta-analysis (Title/Abstract)
#31	Overview (Title/Abstract)
#32	Literature review (Title/Abstract)
#33	Rapid review (Title/Abstract)
#34	Umbrella review (Title/Abstract)
#35	Mapping review (Title/Abstract)
#36	Review (Title/Abstract)
#37	#29 OR #30 OR #31 OR #32 OR #33 OR #34 OR #35 OR #36
#38	#3 OR #28 AND #37

Supplementary material Table 2 Specific items in AMSTAR-2

Specific item information	Results
1. Did the research questions and inclusion criteria for the review include the components of PICO?	Y/N
2. Did the report of the review contain an explicit statement that the review methods were established prior to the conduct of the review and did the report justify any significant deviations from the protocol?	Y/PY/N
3. Did the review authors explain their selection of the study designs for inclusion in the review?	Y/N
4. Did the review authors use a comprehensive literature search strategy?	Y/PY/N
5. Did the review authors perform study selection in duplicate?	Y/N
6. Did the review authors perform data extraction in duplicate?	Y/N
7. Did the review authors provide a list of excluded studies and justify the exclusions?	Y/PY/N
8. Did the review authors describe the included studies in adequate detail?	Y/PY/N
9. Did the review authors use a satisfactory technique for assessing the risk of bias (RoB) in individual studies that were included in the review?	Y/PY/N
10. Did the review authors report on the sources of funding for the studies included in the review?	Y/N
11. If meta-analysis was performed, did the review authors use appropriate methods for statistical combination of results?	Y/N/No meta-analysis conducted
12. If meta-analysis was performed, did the review authors assess the potential impact of RoB in individual studies on the results of the meta-analysis or other evidence synthesis?	Y/N/No meta-analysis conducted
13. Did the review authors account for RoB in primary studies when interpreting/discussing the results of the review?	Y/N
14. Did the review authors provide a satisfactory explanation for, and discussion of, any heterogeneity observed in the results of the review?	Y/N
15. If they performed quantitative synthesis did the review authors carry out an adequate investigation of publication bias (small study bias) and discuss its likely impact on the results of the review?	Y/N/No meta-analysis conducted
16. Did the review authors report any potential sources of conflict of interest, including any funding they received for conducting the review?	Y/N

Notes: Y=yes; N=no; PY= Partial yes.

Refer to Shea BJ, Reeves BC, Wells G, Thuku M, Hamel C, Moran J, et al. AMSTAR 2: a critical appraisal tool for systematic reviews that include randomised or non-randomised studies of healthcare interventions, or both. BMJ. 2017;358:j4008. Published 2017 Sep 21. doi:10.1136/bmj.j4008

Supplementary material Table 3 Basic characteristics and reasons for the withdrawal in eight Cochrane reviews

Title	Year of Protocol	Is Protocol updated?	Is the review published in full?	Is the review updated?	Published and Version	Reason for withdrawal	Website link of withdrawal
Case management for persons with substance use disorders	2006	N	Y	Y	2007 version 1<br>2014 version 2	the authors are not available for updating it	https://www.cochranelibrary.com/cdsr/doi/10.1002/14651858.CD006265.pub3/full
Interventions for preventing tobacco smoking in public places	N	N	Y	Y	2000 version 1<br>2008 version 2	lack recent evidence	https://www.cochranelibrary.com/cdsr/doi/10.1002/14651858.CD001294.pub2/full
Interventions for smoking cessation in indigenous populations	2011	N	Y	Y	2012 version 1<br>2021 version 2	lack recent evidence; not reflect up-to-date standards.	https://www.cochranelibrary.com/cdsr/doi/10.1002/14651858.CD009046.pub3/full
Interventions for tobacco use prevention in indigenous youth	2011	N	Y	Y	2012 version 1<br>2021 version 2	lack recent evidence; not reflect up-to-date standards.	https://www.cochranelibrary.com/cdsr/doi/10.1002/14651858.CD009325.pub3/full
Pharmacotherapy for smoking cessation: effects by subgroup defined by genetically informed biomarkers	2015	N	Y	Y	2017 version 1<br>2021 version 2	non-compliant with the commercial sponsorship policy	https://www.cochranelibrary.com/cdsr/doi/10.1002/14651858.CD011823.pub3/full

Title	Year of Protocol	Is Protocol updated?	Is the review published in full?	Is the review updated?	Published and Version	Reason for withdrawal	Website link of withdrawal
Psychosocial interventions for Cocaine and psychostimulant amphetamines related disorders	2001	N	Y	Y	2007 version 1<br>2015 version 2	non-compliant with the commercial sponsorship policy;<br>new author team will update and replace this review.	https://www.cochranelibrary.com/cdsr/doi/10.1002/14651858.CD003023.pub3/full
Quit and Win contests for smoking cessation	2004	N	Y	Y	2005 version 1<br>2008 version 2<br>2021 version 3	be superseded by “competitions for smoking cessation”	https://www.cochranelibrary.com/cdsr/doi/10.1002/14651858.CD004986.pub4/full
Reduction versus abrupt cessation in smokers who want to quit	2009	N	Y	Y	2010 version 1<br>2012 version 2<br>2019 version 3	be superseded by “smoking reduction interventions for smoking cessation”	https://www.cochranelibrary.com/cdsr/doi/10.1002/14651858.CD008033.pub4/full

Notes: Y=Yes; N=No

Supplementary material Table 4 Summary of interventions of the 71 included Cochrane reviews

Type of intervention		Point time of intervention	Length of intervention	Potential mechanism of intervention						Safety evaluation	Number of reviews	Study ID
				Enhance motivation	Efficacy of smoking cessation	Reduces relapse rate	Develop new habits	Relieves nicotine abstinence reaction	Build a smoke-free environment			
Western medicine therapy	Nicotine Replacement Therapy（NRT）	1w/2w/3w/8w/26w/28w/1m/2m/3m/6m/7m/9m/11m/12m	1w-12m	N	Y	Y	N	Y	N	Y	10	Lindson-Hawley 2016[63]; Hartmann-Boyce 2018[5]; Claire 2020[30]; Cahill 2013[23]; Fanshawe 2017[35]; Annika 2023[68]; Thordis 2014[80]; Jon 2014[52]; Jonathan 2018[53]; Eva 2016[34]
	Varenicline	1w/3w/7w/11w/28w/34w/52w/2m/11m/12m/15m	3w-15m	Y	Y	Y	N	Y	N	Y	8	Jonathan 2023[25]; Cahill 2013[23]; Thordis 2014[80]; Jon 2014[52]; Daniel 2012[31]; Streck 2024[67]; Jonathan 2018[53]; Eva 2016[34]
	Bupropion	1w/3w/6w/11w/26w/34w/2m/3m/6m/7m/11m/12m/15m/	1w-15m	Y	Y	Y	N	Y	N	Y	8	Claire 2020[30]; Cahill 2013[23]; Daniel 2012[31]; Streck 2024[67]; Jonathan 2018[53]; Eva 2016[34]; Regina 2013[71]; Anisa 2023[47]

Type of intervention	Point time of intervention	Length of intervention	Potential mechanism of intervention: Enhance motivation	Efficacy of smoking cessation	Reduces relapse rate	Develop new habits	Relieves nicotine abstinence reaction	Build a smoke-free environment	Safety evaluation	Number of reviews	Study ID
Nortriptyline	1w/6w/26w/12m	1w-12m	N	Y	N	N	Y	N	Y	2	Eva 2016[34]; Anisa 2023[47]
Propofol	2m/11m	2-11m	Y	N	N	N	Y	N	Y	1	Jon 2014[52]
Nicobrevin	10w/12m	10w-12m	N	N	N	N	Y	N	N	1	Stead 2006[75]
Cytisine	7w/52w	7-52w	N	Y	Y	N	Y	N	Y	1	Jonathan 2023[25]
Dianicline	7w/52w	7-52w	N	Y	Y	N	Y	N	Y	1	Jonathan 2023[25]
Nicotine vaccines	1m/4m	1-4m	N	Y	Y	N	Y	N	Y	1	Hartmann-Boyce 2012[42]
Naltrexone	4w/27w	4-27w	N	Y	N	N	Y	N	Y	1	David 2013[32]
Silver acetate	3w/6w	3-6w	N	Y	N	N	Y	N	Y	1	Tim 2012[81]
Monoamine oxidase inhibitor (MAOI)	6w/26w	6-26w	N	N	N	N	Y	N	Y	1	Anisa 2023[47]
Selective serotonin reuptake inhibitors (SSRIs)	6w/26w	6-26w	N	N	N	N	Y	N	Y	1	Anisa 2023[47]
Hypericum (St John's wort)	6w/26w	6-26w	N	N	N	N	Y	N	Y	1	Anisa 2023[47]
Buspirone	3w/12w	3-12w	N	N	N	N	Y	N	N	1	Hughes 2000[48]
Diazepam	3w/12w	3-12w	N	N	N	N	Y	N	N	1	Hughes 2000[48]
Meprobamate	3w/12w	3-12w	N	N	N	N	Y	N	N	1	Hughes 2000[48]
Beta-blockers	3w/12w	3-12w	N	N	N	N	Y	N	N	1	Hughes 2000[48]
Rimonabant	12w/42w	12-42w	N	N	Y	N	Y	N	Y	1	Cahill 2011[22]

	Type of intervention	Point time of intervention	Length of intervention	Potential mechanism of intervention: Enhance motivation	Efficacy of smoking cessation	Reduces relapse rate	Develop new habits	Relieves nicotine abstinence reaction	Build a smoke-free environment	Safety evaluation	Number of reviews	Study ID
	Clonidine	4w/52w	4-52w	N	N	N	N	Y	N	Y	1	Gourlay 2004[39]
	Pharmacotherapy (not classified)	6w/3m/18m/24m	1w-24m	N	Y	Y	N	Y	N	N	3	Apollonio 2016[18]; Cahill 2014[24]; Eva 2016[34]
	E-cigarette (including Heated tobacco)	2w/3w/8w/13w/6m/12m	2w-12m	N	Y	N	Y	N	N	Y	3	Lindson-Hawley 2016[63]; Jamie 2022[50]; Harry 2021[41]
Non-pharmacological therapy	Counseling（including individual counselling, Group counselling, Face-to-face counselling, Tailored telephone counseling, Front-loaded counselling etc.）	1m/6m	1-6m	Y	Y	N	N	N	N	N	7	Fanshawe 2017[35]; Cahill 2014[24]; Tim 2016[82]; Apollonio 2016[18]; Catherine 2016[28]; William 2019[87]; Flora 2018[37]
	Behavioural intervention	1d/1w/6m/12m/104w	6-12m	Y	Y	Y	N	N	N	N	5	Taghrid 2023[64]; Thordis 2014[80]; Eva 2016[34]; Hartmann-Boyce 2019[44]; Hartmann-Boyce 2021[45]
	Incentives（cash, a prize draw etc.）	27w/11.5m	27w-11.5m; 192d	Y	N	N	N	N	N	N	2	Caitlin 2018[27]; Catherine 2016[28]
	Behavioural reduction advice	6m/12m/36m	6-12m	Y	N	N	N	N	N	N	2	Lindson-Hawley 2016[63]; Stead 2013[77]
	Psychosocial intervention	10w/12w	10 w	Y	N	N	N	N	N	N	2	Jürgen 2014[55]; Van 2003[83]

Type of intervention	Point time of intervention	Length of intervention	Potential mechanism of intervention: Enhance motivation	Efficacy of smoking cessation	Reduces relapse rate	Develop new habits	Relieves nicotine abstinence reaction	Build a smoke-free environment	Safety evaluation	Number of reviews	Study ID
Exercise	6m/11.5m/16m	6-16m	Y	Y	N	Y	N	N	N	2	Michael 2019[66]; Catherine 2016[28]
Motivational interviewing	4w/16w	4-16w	Y	Y	N	N	N	N	N	1	Lindson 2019[61]
Feedback	11.5m	11.5m	Y	N	N	N	N	N	N	1	Catherine 2016[28]
Health education	11.5m	11.5m	Y	N	N	N	N	N	N	1	Catherine 2016[28]
Smoking cessation advice	NR	NR	Y	Y	N	N	N	N	Y	1	Khanna 2016[56]
Reduction to quit	1w/18m	1w-18m	NR	N	N	N	N	N	N	1	Lindson 2019[61]
Competitions	1m/6m	1-6m	Y	Y	N	N	N	N	Y	1	Thomas 2019[79]
Group behavioural therapy	3m/24m	3-24m	Y	Y	Y	N	N	N	N	1	Cahill 2014[24]
Stage-based interventions	6m/24m	6-24m	Y	N	N	N	N	N	N	1	Cahill 2010[26]
Text messaging	1w/6m	1w-6m	Y	N	N	N	N	N	N	1	Whittaker 2019[86]
Tailored Internet intervention	2m	2m	Y	N	N	N	N	N	N	1	Gemma 2016[38]
Print-based self-help interventions（e.g written materials, videos, audiotapes）	55d	55d	Y	N	N	N	N	N	N	1	Jonathan 2018[54]
The ABC pathway（ask, brief advice, and cessation support）	2m/3m	2-3m	Y	N	N	N	N	N	Y	1	Roelsgaard 2019[74]

	Type of intervention	Point time of intervention	Length of intervention	Potential mechanism of intervention						Safety evaluation	Number of reviews	Study ID
				Enhance motivation	Efficacy of smoking cessation	Reduces relapse rate	Develop new habits	Relieves nicotine abstinence reaction	Build a smoke-free environment			
	aversion method	2w/8w	2-8w	N	N	Y	N	N	N	N	1	Hajek 2001[40]
	Biomedical risk assessment	6m/12m	6-12m	Y	N	N	N	N	N	N	1	Clair 2019[29]
	Mindfulness training	6m	8w	N	N	N	N	N	N	N	1	Jackson 2022[49]
	Hypnotherapy	2w/48w	2-48w	Y	Y	N	N	N	N	N	1	Joanne 2019[51]
	Low-nicotine cigarettes	6m/12m	6-12m	N	N	N	Y	N	N	Y	1	Lindson-Hawley 2016[63]
	Snus	6m/12m	6-12m	N	N	N	Y	N	N	Y	1	Lindson-Hawley 2016[63]
	Electronic Health Record (HER)	6m/19m	6-19m	Y	N	N	N	N	N	N	1	Boyle 2014[21]
Mass media	Mass media（including branded tobacco packaging）	6m/18y	6m-18y	Y	N	N	N	N	N	Y	2	Bala 2017[20]; McNeill 2017[65]
Tradition-al Chinese medicine therapy	Acupuncture（including Acupressure, Electrostimulation）	20min/12w	20min-12w	N	N	Y	N	Y	N	N	1	White 2014[85]
	Auricular stimulation	20min/12w	20min-12w	N	N	Y	N	Y	N	N	1	White 2014[85]
Policy	Healthcare financing systems（including cost-free medications）	2w/26w	2-26w	Y	Y	N	N	N	N	N	2	Floor 2017[36]; Nicola 2021[69]
	System change interventions	6m/24m	6-24m	Y	Y	N	N	N	N	N	1	Dennis 2017[33]

	Type of intervention	Point time of intervention	Length of intervention	Potential mechanism of intervention: Enhance motivation	Efficacy of smoking cessation	Reduces relapse rate	Develop new habits	Relieves nicotine abstinence reaction	Build a smoke-free environment	Safety evaluation	Number of reviews	Study ID
Other support	Training of health professionals	6m/48m	6-48m	N	Y	N	N	N	N	N	2	Kristin 2012[57]; Richard 2020[73]
	Social support	3m/11.5m/24m	3-24m	Y	Y	Y	N	N	N	N	2	Cahill 2014[24]; Catherine 2016[28]
	Community pharmacy personnel interventions	6m/12m	6-12m	Y	Y	N	N	N	N	N	1	Kristin 2019[58]
	Group behaviour therapy programmes	6m/60m	6-60m	Y	Y	N	N	N	N	N	1	Lindsay 2017[60]
	Partner support	1m/6m	1-6m	Y	Y	N	N	N	N	N	1	Babalola 2018[19]
	Nursing intervention	14w/26w	14-26w	Y	Y	N	N	N	N	N	1	Rice 2017[72]
Combination therapy	Western medicine therapy + Behavioural intervention	8w/16w/6m/12m	8-12m	N	Y	N	N	N	N	Y	3	Mdege 2024[70]; Taghrid 2023[64]; Jon 2014[52]
	Western medicine therapy + counselling	6w/18m	6w-18m	Y	N	N	N	N	N	N	1	Apollonio 2016[18];
	Bupropion+ counselling	11w/34w	11-34w	Y	N	Y	N	N	N	Y	1	Daniel 2012[31]
	Bupropion + Proactive telephone counselling + individual counselling	10w/12w	10-12w	Y	N	N	N	N	N	N	1	Van 2003[83]
	Psychosocial + pharmacological interventions	10w/12w	10-12w	Y	N	N	N	N	N	N	1	Van 2003[83]
	Psychological + Brief attention (relaxation)/ advice	2w/48w	2-48w	N	Y	N	N	N	N	N	1	Joanne 2019[51]
	Hypnotherapy + Brief attention (relaxation)/	2w/48w	2-48w	N	Y	N	N	N	N	N	1	Joanne 2019[51]

Type of intervention	Point time of intervention	Length of intervention	Potential mechanism of intervention: Enhance motivation	Efficacy of smoking cessation	Reduces relapse rate	Develop new habits	Relieves nicotine abstinence reaction	Build a smoke-free environment	Safety evaluation	Number of reviews	Study ID
advice											
Hypnotherapy + Psychological treatments	2w/48w	2-48w	N	Y	N	N	N	N	N	1	Joanne 2019[51]
Hypnotherapy + Psychological treatments + aversion ?? method	2w/48w	2-48w	N	Y	N	N	N	N	N	1	Joanne 2019[51]
Individual counselling + the use of a self-help cessation manual	10w/12w	10-12w	N	N	N	N	N	N	N	1	Van 2003[83]
Nicotine Replacement Therapy + Bupropion	2m/15m	2-15m	N	N	Y	N	N	N	N	1	Jonathan 2018[54]
Nicotine Replacement Therapy + Hypnotherapy	2w/48w	2-48w	N	Y	N	N	N	N	N	1	Joanne 2019[51]
Nicotine Replacement Therapy + Print-based self-help interventions	55d	55days	N	N	N	N	N	N	N	1	Jonathan 2018[54]
Nicotine Replacement Therapy + Naltrexone	4w/27w	4-27w	N	Y	N	N	N	N	Y	1	David 2013[32]
Nicotine Replacement Therapy + Nortriptyline	6w/26w	6-26w	N	N	N	N	N	N	Y	1	Anisa 2023[47]
Nicotine Replacement Therapy + Selective serotonin reuptake inhibitor (SSRI)	6w/26w	6-26w	N	N	N	N	N	N	Y	1	Anisa 2023[47]
Nicotine patch + Bupropion	3w/28w	3-28w	N	Y	Y	N	N	N	Y	1	Cahill 2013[23]
Mecamylamine plus nicotine patch	4w/6w	4-6w	N	N	N	N	N	N	N	1	Lancaster 2011[59]

Notes: Y=Yes; N=No; d=day; w=week; m=month; NR=Not reported

Supplementary material Table 5 Effects of interventions of the 71 included Cochrane reviews

Smoking cessation interventions	Measurement time points	Comparison	Meta-analysis						Study ID
			Number of included studies	Number of included participants	Effect size (95% CI)	I^2	Quality of evidence	Subgroup analysis	
**Outcome 1: Prolonged abstinence rate**									
Non-pharmaceutical therapy	3-6 m	Face-to-face behavioral intervention vs minimal or no support	5	851	RR 2.64 (1.66 to 4.20)	44%	L	Y	Taghrid 2023[64]
	6 m	Reduction to quit vs. Abrupt quitting	22	9219	RR 1.01 (0.87 to 1.17)	29%	M	Y	Lindson 2019[61]
	6 m	Psychosocial mood management vs. Control for smokers with current depression	11	1844	RR 1.47 (1.13 to 1.92)	0%	L	Y	Regina 2013[71]
	6 m	Psychosocial mood management vs. Control for smokers with past depression	13	1496	RR 1.41 (1.13 to 1.77)	23%	L	Y	Regina 2013[71]
	6 m	Real-time video counselling vs. Telephone counselling	2	608	RR 2.15 (0.38 to 12.14)	66%	VL	Y	Flora 2018[37]
	6 m	Reduction to quit vs. No treatment	6	1599	RR 1.74 (0.90 to 3.38)	45%	VL	Y	Lindson 2019[61]
	12 m	Brief intervention	4	493	RR 0.92 (0.72 to 1.19)	0%	M	Y	Thordis 2014[80]
	12 m	Intensive intervention	2	210	RR 0.42 (0.27 to 0.65)	0%	M	Y	Thordis 2014[80]
	≥6 m	Individual counselling vs. Minimal contact control	27	11100	RR 1.57 (1.40 to 1.77)	50%	H	Y	Tim 2016[82]
	≥6 m	Quitting with incentives vs. Controls at longest follow-up	30	20097	RR 1.49 (1.28 to 1.73)	33%	H	Y	Caitlin 2018[27]
	≥6 m	Counselling compared to no smoking cessation counselling (intensity 4 vs 0)	28	8234	RR 1.36 (1.24 to 1.49)	34%	H	Y	Streck 2024[67]

Smoking cessation interventions	Measurement time points	Comparison	Meta-analysis						Study ID
			Number of included studies	Number of included participants	Effect size (95% CI)	I^2	Quality of evidence	Subgroup analysis	
	≥6 m	Behavioural support vs. Guaranteed financial incentives	19	8877	OR 1.46 (1.15 to 1.85)	NR	H	N	Hartmann-Boyce 2021[45]
	≥6 m	Behavioural support vs. The provision of counselling	194	72273	OR 1.44 (1.22 to 1.70)	NR	H	N	Hartmann-Boyce 2021[45]
	≥6 m	Internet intervention vs. An active control	5	3806	RR 0.92 (0.78 to 1.09)	0%	H	Y	Gemma 2016[38]
	≥6 m	Incentives in pregnant women at longest follow-up	9	2273	RR 2.38 (1.54 to 3.69)	42%	M	Y	Caitlin 2018[27]
	≥6 m	Tailored materials vs. No materials	10	14359	RR 1.34 (1.19 to 1.51)	0%	M	Y	Jonathan 2018[54]
	≥6 m	Counselling compared to no smoking cessation counselling (intensity 2 vs 0)	12	4432	RR 1.27 (1.02 to 1.58)	54%	M	Y	Streck 2024[67]
	≥6 m	Behavioral treatment vs. No treatment or usual care	1	3562	RR 25.38 (8.03 to 80.22)	NA	M	Y	Eva 2016[34]
	≥6 m	All nursing intervention vs. Control trials	44	20881	RR 1.29 (1.21 to 1.38)	50%	M	Y	Rice 2017[72]
	≥6 m	High-intensity nursing intervention vs. Control trials	37	16865	RR 1.29 (1.21 to 1.38)	53%	M	Y	Rice 2017[72]
	≥6 m	More intensive vs. Less intensive counselling	11	2920	RR 1.29 (1.09 to 1.53)	48%	M	Y	Tim 2016[82]
	≥6 m	Low-intensity nursing intervention vs. Control trials	7	4016	RR 1.27 (0.99 to 1.62)	36%	M	Y	Rice 2017[72]

Smoking cessation interventions	Measurement time points	Comparison	Meta-analysis						Study ID
			Number of included studies	Number of included participants	Effect size (95% CI)	I^2	Quality of evidence	Subgroup analysis	
	≥6 m	Comparisons between counselling approaches of similar intensity	6	2662	RR 1.24 (1.01 to 1.51)	0%	M	Y	Tim 2016[82]
	≥6 m	Non-tailored self-help (control group given no materials) vs. No self-help	11	13241	RR 1.19 (1.03 to 1.37)	0%	M	Y	Jonathan 2018[54]
	≥6 m	Distress tolerance training vs less intensive smoking cessation treatment	1	49	RR 1.63 (0.33 to 8.08)	NA	L	Y	Jackson 2022[49]
	≥6 m	Yoga vs matched intensive smoking cessation treatment	1	55	RR 1.44 (0.40 to 5.16)	NA	L	Y	Jackson 2022[49]
	≥6 m	Group counselling vs. Control	9	1910	RR 1.35 (1.03 to 1.77)	0%	L	N	Fanshawe 2017[35]
	≥6 m	Acceptance and commitment therapy (ACT) vs NRT	1	102	RR 1.27 (0.53 to 3.02)	NA	L	Y	Jackson 2022[49]
	≥6 m	Motivational interviewing vs. Another smoking cessation intervention	19	5192	RR 1.24 (0.91 to 1.69)	54%	L	Y	Lindson 2019[62]
	≥6 m	Higher intensity motivational interviewing vs. Lower intensity motivational interviewing	5	5620	RR 1.23 (1.11 to 1.37)	0%	L	Y	Lindson 2019[62]
	≥6 m	Interventions using messaging vs. Control	3	2985	RR 1.18 (0.90 to 1.56)	0%	L	N	Fanshawe 2017[35]
	≥6 m	Non-tailored internet-based messages vs. Tailored messages	3	4040	RR 1.17 (0.97 to 1.41)	57%	L	Y	Gemma 2016[38]

Smoking cessation interventions	Measurement time points	Comparison	Meta-analysis						Study ID
			Number of included studies	Number of included participants	Effect size (95% CI)	I^2	Quality of evidence	Subgroup analysis	
	≥6 m	A tailored and interactive internet intervention vs. A non-active control	9	7909	RR 1.15 (1.02 to 1.30)	53%	L	Y	Gemma 2016[38]
	≥6 m	Behavioural support vs. brief advice or no intervention	7	2314	RR 1.11 (0.87 to 1.42)	0%	L	Y	Mdege 2024 [70]
	≥6 m	Individual counselling vs. Control	7	2088	RR 1.07 (0.83 to 1.39)	1%	L	N	Fanshawe 2017[35]
	≥6 m	Acceptance and commitment therapy (ACT) vs less intensive ACT	1	100	RR 1.00 (0.50 to 2.01)	NA	L	Y	Jackson 2022[49]
	≥6 m	Mindfulness training compared with matched intensity smoking cessation treatment	3	542	RR 0.99 (0.67 to 1.46)	0%	L	Y	Jackson 2022[49]
	≥6 m	Distress tolerance training vs matched-intensity	1	69	RR 0.87 (0.26 to 2.98)	NA	L	Y	Jackson 2022[49]
	≥6 m	Motivational interviewing vs. No treatment for smoking cessation	4	684	RR 0.84 (0.63 to 1.12)	0%	L	Y	Lindson 2019[62]
	≥6 m	Mindfulness training vs no treatment	1	352	RR 0.81 (0.43 to 1.53)	NA	L	Y	Jackson 2022[49]
	≥6 m	Computer-based interventions vs. Control	3	340	RR 0.79 (0.50 to 1.24)	0%	L	N	Fanshawe 2017[35]
	≥6 m	Single session behavioural support vs. Usual care, brief advice, or very brief advice	4	6328	RR 1.86 (1.01 to 3.41)	66%	VL	Y	Richard 2020[73]
	≥6 m	Counselling compared to no smoking cessation counselling (intensity 1 vs 0)	2	1417	RR 1.52 (0.80 to 2.89)	74%	VL	Y	Streck 2024[67]

Smoking cessation interventions	Measurement time points	Comparison	Meta-analysis						Study ID
			Number of included studies	Number of included participants	Effect size (95% CI)	I^2	Quality of evidence	Subgroup analysis	
	≥6 m	Behavioural support from dental professional at high school/college vs. Usual care/no inter_x005fvention	3	1020	RR 1.51 (0.86 to 2.65)	83%	VL	Y	Richard 2020[73]
	≥6 m	Health system telephone counselling vs. quitline	3	3260	RR 1.23 (1.00 to 1.51)	20%	VL	Y	Streck 2024[67]
	≥6 m	Quitline referral vs. control	2	1870	RR 1.17 (0.70 to 1.96)	71%	VL	Y	Streck 2024[67]
	≥6 m	Counselling compared to no smoking cessation counselling (intensity 3 vs 0)	7	4627	RR 1.04 (0.90 to 1.20)	7%	VL	Y	Streck 2024[67]
	6-48 m	Training vs. Control group	8	9443	OR 1.60 (1.26 to 2.03)	59%	M	Y	Kristin 2012[57]
Pharmacotherapy	6 m	Bupropion vs. Control for smokers with past depression	4	404	RR 2.04 (1.31 to 3.18)	44%	L	Y	Regina 2013[71]
	6 m	Bupropion vs. Control for smokers with current depression	5	410	RR 1.37 (0.83 to 2.27)	29%	L	Y	Regina 2013[71]
	12 m	Varenicline vs. Placebo	2	1297	RR 1.23 (1.08 to 1.41)	0%	M	Y	Jonathan 2018[53]
	6-12 m	Nicotine EC compared to NRT	6	2378	RR 1.63 (1.30 to 2.04)	10%	H	Y	Jamie 2022[50]
	6-12 m	Nicotine EC compared to non-nicotine EC	5	1447	RR 1.94 (1.21 to 3.13)	0%	M	Y	Jamie 2022[50]
	6-12 m	Nicotine EC compared to behavioural support only/no support	7	3126	RR 2.66 (1.52 to 4.65)	0%	VL	Y	Jamie 2022[50]
	≥6 m	Pharmacological treatment (varenicline) vs. Placebo	1	504	RR 3.34 (1.88 to 5.92)	NA	H	Y	Eva 2016[34]

Smoking cessation interventions	Measurement time points	Comparison	Meta-analysis						Study ID
			Number of included studies	Number of included participants	Effect size (95% CI)	I^2	Quality of evidence	Subgroup analysis	
	≥6 m	Pharmacological treatment (NRT) vs. Placebo	1	370	RR 2.60 (1.29 to 5.24)	NA	H	Y	Eva 2016[34]
	≥6 m	Pharmacological treatment vs. Placebo	4	1429	RR 2.53 (1.83 to 3.50)	0%	H	Y	Eva 2016[34]
	≥6 m	varenicline vs placebo	41	17395	RR 2.32 (2.15 to 2.51)	60%	H	Y	Jonathan 2023[25]
	≥6 m	Bupropion vs. placebo/no pharmacological treatment	50	18577	RR 1.60 (1.49 to 1.72)	16%	H	Y	Anisa 2023[47]
	≥6 m	Any type of NRT vs. Placebo/no NRT control	133	64640	RR 1.55 (1.49 to 1.61)	39%	H	Y	Hartmann-Boyce 2018[43]
	≥6 m	Varenicline vs. bupropion	9	7560	RR 1.36 (1.25 to 1.49)	0%	H	Y	Jonathan 2023[25]
	≥6 m	NRT vs. placebo or no NRT	8	3838	RR 1.33 (1.05 to 1.67)	43%	H	Y	Streck 2024[67]
	≥6 m	Varenicline vs. nicotine replacement therapy monotherapy	11	7572	RR 1.25 (1.14 to 1.37)	28%	H	Y	Jonathan 2023[25]
	≥6 m	Pharmacological treatment (bupropion) vs. Placebo	2	503	RR 2.03 (1.26 to 3.28)	NA	M	Y	Eva 2016[34]
	≥6 m	Pharmacological treatment vs. A different pharmacological treatment	1	543	RR 2.50 (1.47 to 4.26)	NA	M	Y	Eva 2016[34]
	≥6 m	Varenicline vs. placebo	2	427	RR 1.95 (1.05 to 3.62)	0%	M	Y	Mdege 2024 [70]
	≥6 m	cytisine vs. placebo	4	4623	RR 1.30 (1.15 to 1.47)	83%	M	Y	Jonathan 2023[25]
	≥6 m	Varenicline vs. placebo or no varenicline	4	829	RR 1.29 (0.96 to 1.75)	49%	M	Y	Streck 2024[67]
	≥6 m	cytisine vs. varenicline	2	2131	RR 0.83 (0.66 to 1.05)	0%	M	Y	Jonathan 2023[25]

Smoking cessation interventions	Measurement time points	Comparison	Meta-analysis						Study ID
			Number of included studies	Number of included participants	Effect size (95% CI)	I^2	Quality of evidence	Subgroup analysis	
	≥6 m	Pharmacological treatment (nortriptyline) vs. Placebo	1	100	RR 2.54 (0.87 to 7.44)	NA	L	Y	Eva 2016[34]
	≥6 m	Cytisine vs. nicotine replacement therapy	1	1310	RR 1.43 (1.13 to 1.80)	NA	L	Y	Jonathan 2023[25]
	≥6 m	Bupropion vs. placebo or no bupropion	4	872	RR 1.11 (0.86 to 1.43)	6%	L	Y	Streck 2024[67]
	≥6 m	Varenicline vs. combination nicotine replacement therapy	5	2344	RR 1.02 (0.87 to 1.20)	0%	L	Y	Jonathan 2023[25]
	≥6 m	Varenicline vs. e-cigarettes	1	54	RR 3.25 (1.21 to 8.71)	NA	VL	Y	Jonathan 2023[25]
	≥6 m	Bupropion vs. Placebo	1	207	RR 1.49 (0.55 to 4.02)	NA	VL	N	Fanshawe 2017[35]
	≥6 m	Cytisine vs. NRT	1	200	RR 1.18 (0.66 to 2.11)	NA	VL	Y	Mdege 2024 [70]
	≥6 m	NRT vs. Placebo	2	385	RR 1.11 (0.48 to 2.58)	20%	VL	N	Fanshawe 2017[35]
	≥6 m	Varenicline vs. NRT	1	200	RR 0.93 (0.48 to 1.83)	NA	VL	N	Mdege 2024 [70]
Combination therapy	6 m	Combined pharmacotherapy and behavioral interventions vs. brief advice or usual care	52	19488	RR 1.83 (1.68 to 1.98)	36%	H	Y	Stead 2016[78]
	≥6 m	Behavioural intervention plus NRT/e-cigarette vs. No intervention/usual care, brief advice, or very brief advice	4	1221	RR 2.76 (1.58 to 4.82)	0%	M	Y	Richard 2020[73]
	≥6 m	An internet programme plus behavioural support vs. A non-active control	5	2334	RR 1.69 (1.30 to 2.18)	60%	M	Y	Gemma 2016[38]
	≥6 m	Bupropion plus varenicline vs. varenicline	3	1057	RR 1.21 (0.95 to 1.55)	15%	M	Y	Anisa 2023[47]

Smoking cessation interventions	Measurement time points	Comparison	Meta-analysis						Study ID
			Number of included studies	Number of included participants	Effect size (95% CI)	I^2	Quality of evidence	Subgroup analysis	
	≥6 m	An interactive or tailored internet intervention, or both vs. An internet intervention that was not tailored/interactive.	7	14623	RR 1.10 (0.99 to 1.22)	0%	M	Y	Gemma 2016[38]
	≥6 m	The internet plus behavioural support vs. Active control	4	2769	RR 1.00 (0.84 to 1.18)	0%	M	Y	Gemma 2016[38]
	6 m	Reduction and pharmacotherapy vs. Reduction to quit alone (placebo or no pharmacotherapy)	11	8636	RR 1.68 (1.09 to 2.58)	78%	L	Y	Lindson 2019[61]
	≥6 m	Behavioural support + NRT vs. behavioural support alone	1	560	RR 1.47 (0.92 to 2.36)	NA	L	Y	Mdege 2024 [70]
	≥6 m	Bupropion plus nicotine replacement therapy (NRT) vs. NRT	15	4117	RR 1.17 (0.95 to 1.44)	43%	L	Y	Anisa 2023[47]
	≥6 m	Motivational interviewing in addition to other smoking cessation treatment vs. Other smoking cessation treatment alone	12	4167	RR 1.07 (0.85 to 1.36)	47%	L	Y	Lindson 2019[62]
	≥6 m	Behavioural support + NRT vs. brief advice	1	15	RR 8.00 (0.51 to 126.67)	NA	VL	Y	Mdege 2024 [70]
	≥6 m	Bupropion therapy plus behavioral support vs. self-help	1	86	RR 1.94 (0.94 to 4.00)	NA	VL	Y	Taghrid 2023[64]

Smoking cessation interventions	Measurement time points	Comparison	Meta-analysis						Study ID
			Number of included studies	Number of included participants	Effect size (95% CI)	I^2	Quality of evidence	Subgroup analysis	
	≥6 m	Multi-session behavioural support vs. Usual care, brief advice, or very brief advice, or less active treatment	7	2639	RR 1.90 (1.17 to 3.11)	61%	VL	Y	Richard 2020[73]
	≥6 m	Mindfulness-based relapse prevention vs no treatment	1	86	RR 1.43 (0.56 to 3.67)	NA	VL	Y	Jackson 2022[49]
	≥6 m	Acceptance and commitment therapy (ACT) vs brief advice	1	144	RR 1.27 (0.59 to 2.75)	NA	VL	Y	Jackson 2022[49]
	≥6 m	Interventions with multiple delivery methods vs. Control vs control	8	2755	RR 1.26 (0.95 to 1.66)	0%	VL	N	Fanshawe 2017[35]
	≥6 m	Mindfulness training vs. less intensive	5	813	RR 1.19 (0.65 to 2.19)	60%	VL	Y	Jackson 2022[49]
	≥6 m	Nicotine patch plus bupropion vs. Nicotine patch plus placebo	1	211	RR 1.05 (0.41 to 2.69)	NA	VL	N	Fanshawe 2017[35]
	≥6 m	Bupropion therapy plus behavioral intervention vs. behavioral intervention alone	1	121	RR 0.77 (0.42 to 1.41)	NA	VL	Y	Taghrid 2023[64]
**Outcome 2: Repeated point-prevalence abstinence rate**									
Non-pharmaceutical therapy	<3 m	Contingent reinforcement in addition to usual care vs. Usual care	1	120	RR 0.67 (0.16 to 2.77)	NA	L	N	Vijayaraghavan 2020[84]
	< 6 m	Medication adherence intervention plus standard care vs. Standard care alone	5	1795	RR 1.08 (0.96 to 1.21)	0%	L	Y	Hollands 2019[46]

Smoking cessation interventions	Measurement time points	Comparison	Meta-analysis						Study ID
			Number of included studies	Number of included participants	Effect size (95% CI)	I^2	Quality of evidence	Subgroup analysis	
	3-6 m	More intensive behavioral support vs. Less intensive behavioral support	3	657	RR 1.64 (1.01 to 2.69)	0%	VL	N	Vijayaraghavan 2020[84]
	6-12 m	Text messaging vs. Minimal smoking cessation support (all randomized)	13	14133	RR 1.54 (1.19 to 2.00)	71%	M	Y	Whittaker 2019[86]
	6-24 m	Behavioural reduction advice vs. Health mailings	1	320	RR 1.49 (0.59 to 3.76)	NA	VL	Y	Lindson-Hawley 2016[63]
	6-24 m	Low-nicotine cigarettes vs. Regular cigarettes	1	135	RR 1.38 (0.13 to 14.79)	NA	VL	Y	Lindson-Hawley 2016[63]
	≥6 m	Any self-help intervention	6	1906	OR 1.16 (0.74 to 1.82)	0%	H	N	Cahill 2014[24]
	≥6 m	Individual counselling	8	3516	OR 1.96 (1.51 to 2.54)	24%	M	N	Cahill 2014[24]
	≥6 m	Group behavioural therapy	8	1309	OR 1.71 (1.05 to 2.80)	15%	M	N	Cahill 2014[24]
	≥6 m	Interventions to increase adherence through providing information and facilitating problem-solving vs. Behavioural support for smoking cessation	7	3593	RR 1.16 (0.96 to 1.40)	47%	L	Y	Hollands 2019[46]
	≥6 m	Performance-based eligibility competitions vs. Alternative cessation	6	3201	RR 1.16 (0.77 to 1.74)	57%	VL	Y	Thomas 2019[79]
	≥6 m	Multi-issue support vs. Smoking cessation support only	2	146	RR 0.95 (0.35 to 2.61)	25%	VL	N	Vijayaraghavan 2020[84]

Smoking cessation interventions	Measurement time points	Comparison	Meta-analysis						Study ID
			Number of included studies	Number of included participants	Effect size (95% CI)	I^2	Quality of evidence	Subgroup analysis	
Pharmacotherapy	6-24 m	Snus vs. Placebo	1	319	RR 3.06 (0.84 to 11.08)	NA	L	Y	Lindson-Hawley 2016[63]
	6-24 m	E-cigarettes vs. Placebo	1	300	RR 2.75 (0.97 to 7.76)	NA	L	Y	Lindson-Hawley 2016[63]
	6-24 m	Varenicline vs. Placebo	1	218	RR 1.95 (0.86 to 4.40)	NA	L	Y	Lindson-Hawley 2016[63]
	6-24 m	NRT vs. Placebo	8	3081	RR 1.87 (1.43to 2.44)	30%	L	Y	Lindson-Hawley 2016[63]
	6-24 m	Bupropion vs. Placebo	1	594	RR 1.27 (0.67 to 2.40)	NA	L	Y	Lindson-Hawley 2016[63]
	≥6 m	Pharmacological treatments	5	1092	OR 1.98 (1.26 to 3.11)	19%	H	N	Cahill 2014[24]
**Outcome 3: Point-prevalence abstinence rate**									
Non-pharmaceutical therapy	0-4 w	Brief behavioural intervention	7	1141	RR 1.30 (1.16 to 1.46)	75%	H	Y	Thordis 2014[80]
	0-4 w	Behavioural intervention vs. Control	2	210	RR 10.76 (4.55 to 25.46)	0%	M	Y	Thordis 2014[80]
	6 w-18 m	Counselling vs. Usual care or no intervention	11	1759	RR 1.33 (0.90 to 1.95)	28%	L	Y	Apollonio 2016[18]
	6-7 m	More vs. Less intensive social support	6	1614	RR 2.3 (1.33 to 3.97)	54%	L	N	Kristin 2019[58]
	6-16 m	Exercise and smoking cessation support or Exercise alone vs. Smoking cessation support only	21	6607	RR 1.08(0.96 to 1.22)	0%	L	Y	Michael 2019[66]
	6-48 m	Training vs. Control group	14	13459	OR 1.41(1.13 to 1.77)	57%	M	Y	Kristin 2012[57]

Smoking cessation interventions	Measurement time points	Comparison	Meta-analysis						Study ID
			Number of included studies	Number of included participants	Effect size (95% CI)	I^2	Quality of evidence	Subgroup analysis	
	9-15 m	Behavioural interventions for relapse prevention	11	5523	RR 0.98 (0.87 to 1.11)	52%	M	Y	Jonathan 2018[54]
	≥6 m	Group programme vs. Self-help programme	13	4395	RR 1.88 (1.52 to 2.33)	0%	M	Y	Lindsay 2017[60]
	≥6 m	Additional proactive calls vs. Control	14	32484	RR 1.38 (1.19 to 1.61)	72%	M	Y	William 2019[87]
	≥6 m	Cost-free medications plus standard or multicomponent smoking cessation support vs. Standard or multicomponent smoking cessation support	10	7560	RR 1.36 (1.05 to 1.76)	63%	M	N	Nicola 2021[69]
	≥6 m	Adjunctive counseling plus standard or multicomponent smoking cessation support vs. Standard or multicomponent smoking cessation support	22	18150	RR 1.31 (1.10 to 1.55)	44%	M	Y	Nicola 2021[69]
	≥6 m	Tailored print materials plus standard smoking cessation support vs. Standard smoking cessation support	6	15978	RR 1.29 (1.04 to 1.59)	37%	M	N	Nicola 2021[69]
	≥6 m	Proactive telephone counselling vs. Control	65	41233	RR 1.25 (1.15 to 1.35)	52%	M	Y	William 2019[87]
	≥6 m	Group programme plus pharmacotherapy vs. Pharmacotherapy and brief support alone	5	1523	RR 1.11 (0.93 to 1.33)	0%	M	Y	Lindsay 2017[60]
	≥6 m	Group programme vs. Face-to-face individual intervention	6	980	RR 0.99 (0.76 to 1.28)	9%	M	Y	Lindsay 2017[60]

Smoking cessation interventions	Measurement time points	Comparison	Meta-analysis						Study ID
			Number of included studies	Number of included participants	Effect size (95% CI)	I^2	Quality of evidence	Subgroup analysis	
	≥6 m	Group programme vs. No 'intervention’controls	9	1098	RR 2.60 (1.80 to 3.76)	55%	L	Y	Lindsay 2017[60]
	≥6 m	Group programme vs. Brief support	16	7601	RR 1.25 (1.07 to 1.46)	0%	L	Y	Lindsay 2017[60]
	≥6 m	Hypnotherapy vs. Attention-matched behavioural intervention	6	957	RR 1.21 (0.91,1.61)	36%	L	Y	Joanne 2019[51]
	≥6 m	Provider training plus standard or multicomponent smoking cessation support vs. Standard or multicomponent smoking cessation support	7	13685	RR 1.10 (0.85 to 1.41)	66%	L	N	Nicola 2021[69]
	≥6 m	Biomedical feedback plus standard smoking cessation support vs. Standard smoking cessation support	7	3491	RR 1.07 (0.81 to 1.41)	40%	L	N	Nicola 2021[69]
	≥6 m	Cessation interventions with a partner support component vs. Cessation interventions without a partner support component for people who want to quit smoking	7	2573	RR 1.04 (0.88 to 1.22)	0%	L	Y	Babalola 2018[19]
	≥6 m	Cessation interventions with a partner support component vs. Cessation interventions without a partner support	13	2818	RR 0.97 (0.83 to 1.14)	0%	L	Y	Babalola 2018[19]

Smoking cessation interventions	Measurement time points	Comparison	Meta-analysis						Study ID
			Number of included studies	Number of included participants	Effect size (95% CI)	I^2	Quality of evidence	Subgroup analysis	
		component for people who want to quit smoking							
	≥6 m	Hypnotherapy vs. No treatment	1	40	RR 19.0 (1.18, 305.88)	NA	VL	Y	Joanne 2019[51]
	≥6 m	Provider incentives plus standard or multicomponent smoking cessation support vs. Standard or multicomponent smoking cessation support	2	2454	RR 1.14 (0.97 to 1.34)	0%	VL	N	Nicola 2021[69]
	≥6 m	Hypnotherapy vs. Brief behavioural interventions	2	269	RR 0.98 (0.57, 1.69)	0%	VL	Y	Joanne 2019[51]
	≥6 m	Hypnotherapy vs. Intensive behavioural interventions	2	211	RR 0.93 (0.47, 1.82)	0%	VL	Y	Joanne 2019[51]
Pharmacotherapy	6 w-18 m	Pharmacotherapy vs. Usual care or no intervention	11	1808	RR 1.60 (1.22 to 2.12)	56%	L	Y	Apollonio 2016[18]
	12-24 m	Bupropion vs. Placebo	6	1697	RR 1.15 (0.98 to 1.35)	0%	M	Y	Jonathan 2018[54]
	12-15 m	Combination NRT & bupropion vs. Placebo	2	243	RR 1.18 (0.75 to 1.87)	66%	L	Y	Jonathan 2018[54]
	12-15 m	NRT vs. Placebo	2	553	RR 1.04 (0.77 to 1.4)	0%	L	Y	Jonathan 2018[54]
	at the latest time point in pregnancy	NRT vs. Control	9	2336	RR 1.37 (1.08 to 1.74)	34%	L	Y	Claire 2020[30]

Smoking cessation interventions	Measurement time points	Comparison	Meta-analysis						Study ID
			Number of included studies	Number of included participants	Effect size (95% CI)	I^2	Quality of evidence	Subgroup analysis	
Combination therapy	at the latest time point in pregnancy	Bupropion vs. Control	2	76	RR 0.74 (0.21 to 2.64)	0%	L	Y	Claire 2020[30]
	6 w-18 m	Assessed counselling in combination with pharmacotherapy vs. Usual care or no intervention	12	2229	RR 1.74 (1.39 to 2.18)	43%	L	Y	Apollonio 2016[18]
	6 m	Behavioural interventions as adjuncts to pharmacotherapy vs. Pharmacotherapy (NRT)	65	23331	RR 1.15 (1.08 to 1.22)	8%	H	Y	Hartmann-Boyce 2019[44]
Traditional Chinese medicine	6 w-12 m	Acupuncture vs. Sham acupuncture	11	1892	RR 1.10（0.86 to 1.40）	23%	M	Y	White 2014[85]
**Outcome 4：Abstinence rate (the time point and method of measurement were not clear)**									
Non-pharmaceutical therapy	/	Incentives vs. Alternative	4	212	RR 2.36 (1.36 to 4.09)	0%	H	Y	Catherine 2016[28]
	/	Counselling vs. Usual care	30	12432	RR 1.44 (1.19 to 1.73)	49%	H	Y	Catherine 2016[28]
	/	Social support vs. Less intensive interventions	7	781	RR 1.21 (0.93 to 1.58)	54%	H	Y	Catherine 2016[28]
	/	Feedback vs. Usual care	2	355	RR 4.39 (1.89 to 10.21)	0%	M	Y	Catherine 2016[28]
	/	Full financial coverage for abstinence from smoking vs. No coverage	6	9333	RR 1.77 (1.37 to 2.28)	33%	M	Y	Floor 2017[36]

Smoking cessation interventions	Measurement time points	Comparison	Meta-analysis						Study ID
			Number of included studies	Number of included participants	Effect size (95% CI)	I^2	Quality of evidence	Subgroup analysis	
Pharmacotherapy	/	Active dissemination vs. Passive dissemina_x0002_tion	1	194	RR 1.63 (0.62 to 4.32)	NA	M	Y	Catherine 2016[28]
	/	Health education vs. Usual care	5	629	RR 1.59 (0.99 to 2.55)	0%	M	Y	Catherine 2016[28]
	/	Higher-dose compared to lower-dose (21 mg versus 14 mg)	1	537	RR 1.48 (1.06 to 2.08)	NA	M	Y	Annika 2023[68]
	/	Biomedical risk assessment vs. Standard care or minimal intervention for smoking	11	3314	RR 1.26 (0.99 to 1.61)	34%	M	Y	Catherine 2016[28]
	/	Exercise vs. Usual care	1	785	RR 1.20 (0.72 to 2.01)	NA	M	Y	Catherine 2016[28]
	/	Nicotine patch plus a fast-acting form of NRT vs single-form NRT	16	12169	RR 1.27 (1.17 to 1.37)	12%	H	Y	Annika 2023[68]
	/	Higher-dose compared to lower-dose (25 mg versus 15 mg)	3	3446	RR 1.19 (1.00 to 1.41)	0%	M	Y	Annika 2023[68]
	/	Higher-dose compared to lower-dose （42/44 mg versus 21/22 mg）	5	1655	RR 1.09 (0.93 to 1.29)	38%	M	Y	Annika 2023[68]
	/	Biomedical risk assessment vs. Standard care or minimal intervention for smoking	5	2368	RR 1.00 (0.83 to 1.21)	0%	M	Y	Clair 2019[29]
	/	Biomedical risk assessment vs. Standard care or minimal intervention for smoking	5	2064	RR 0.80 (0.63 to 1.01)	0%	L	Y	Catherine 2016[28]

Smoking cessation interventions	Measurement time points	Comparison	Meta-analysis						Study ID
			Number of included studies	Number of included participants	Effect size (95% CI)	I^2	Quality of evidence	Subgroup analysis	
	/	Longer compared to shorter duration of combination NRT （6 weeks versus 2 weeks）	1	987	RR 1.11 (0.94 to 1.31)	NA	L	Y	Annika 2023[68]
	/	Longer compared to shorter duration of combination NRT （16 weeks versus 8 weeks）	1	637	RR 0.96 (0.75 to 1.23)	NA	VL	Y	Annika 2023[68]
**Outcome 5: Adverse events**									
Pharmacotherapy	/	Varenicline vs. placebo or no medication	26	14356	RR 1.23 (1.01 to 1.48)	0%	M	N	Jonathan 2023[25]
	/	Bupropion vs. placebo/no pharmacological treatment	23	10958	RR 1.16 (0.90 to 1.48)	0%	M	N	Anisa 2023[47]
	/	Nicotine EC vs. NRT	4	1702	RR 1.02 (0.88 to 1.19)	0%	M	N	Jamie 2022[50]
	/	Nicotine EC vs. non-nicotine EC	5	840	RR 1.01 (0.91 to 1.11)	0%	M	N	Jamie 2022[50]
	/	Higher-dose vs. lower-dose (42/44 mg versus 21/22 mg)	2	1023	RR 5.01 (0.87 to 28.82)	0%	L	N	Annika 2023[68]
	/	Nicotine patch plus a fast-acting form of NRT vs. single-form NRT	5	2888	RR 4.44 (0.76 to 25.85)	35%	L	N	Annika 2023[68]
	/	Varenicline vs. combination nicotine replacement therapy	4	1852	RR 2.15 (0.49 to 9.46)	0%	L	N	Jonathan 2023[25]
	/	Bupropion vs. placebo/no pharmacological treatment	4	657	RR 1.52 (0.26 to 8.89)	0%	L	N	Anisa 2023[47]

Smoking cessation interventions	Measurement time points	Comparison	Meta-analysis						Study ID
			Number of included studies	Number of included participants	Effect size (95% CI)	I^2	Quality of evidence	Subgroup analysis	
	/	Bupropion plus varenicline vs. varenicline	5	1286	RR 1.23 (0.63 to 2.42)	0%	L	N	Anisa 2023[47]
	/	Nicotine EC vs. behavioural support only/no support	4	765	RR 1.22 (1.12 to 1.32)	41%	L	N	Jamie 2022[50]
	/	Cytisine vs. placebo or no medication	3	3781	RR 1.04 (0.78 to 1.37)	0%	L	N	Jonathan 2023[25]
	/	Varenicline vs. bupropion	5	5317	RR 0.89 (0.61 to 1.31)	0%	L	N	Jonathan 2023[25]
	/	Varenicline vs. nicotine replacement therapy monotherapy	6	6535	RR 0.70 (0.50 to 0.99)	24%	L	N	Jonathan 2023[25]
	/	Cytisine vs. varenicline	2	2017	RR 0.67 (0.44 to 1.03)	45%	L	N	Jonathan 2023[25]
	/	Cytisine vs. nicotine replacement therapy	1	1310	RR 1.15 (0.76 to 1.75)	NR	VL	N	Jonathan 2023[25]

Notes: Y=Yes; N=No; H=High; M=Moderate; L=Low; VL=Very low; NR=Not reported; NA= Not applicable; RR= Relative risk; OR=Odds ratio

Section and Topic	Item #	Checklist item	Location where item is reported
**TITLE**			
Title	1	Identify the report as a systematic review.	Page 1
**ABSTRACT**			
Abstract	2	See the PRISMA 2020 for Abstracts checklist.	Page 1-2
**INTRODUCTION**			
Rationale	3	Describe the rationale for the review in the context of existing knowledge.	Page 3-4
Objectives	4	Provide an explicit statement of the objective(s) or question(s) the review addresses.	Page 4
**METHODS**			
Eligibility criteria	5	Specify the inclusion and exclusion criteria for the review and how studies were grouped for the syntheses.	Page 4-5
Information sources	6	Specify all databases, registers, websites, organisations, reference lists and other sources searched or consulted to identify studies. Specify the date when each source was last searched or consulted.	Page 5-6
Search strategy	7	Present the full search strategies for all databases, registers and websites, including any filters and limits used.	Page 5-6,supple Tab 1
Selection process	8	Specify the methods used to decide whether a study met the inclusion criteria of the review, including how many reviewers screened each record and each report retrieved, whether they worked independently, and if applicable, details of automation tools used in the process.	Page 6
Data collection process	9	Specify the methods used to collect data from reports, including how many reviewers collected data from each report, whether they worked independently, any processes for obtaining or confirming data from study investigators, and if applicable, details of	Page 6-7

Section and Topic	Item #	Checklist item	Location where item is reported
		automation tools used in the process.	
Data items	10a	List and define all outcomes for which data were sought. Specify whether all results that were compatible with each outcome domain in each study were sought (e.g. for all measures, time points, analyses), and if not, the methods used to decide which results to collect.	Page 6-7
	10b	List and define all other variables for which data were sought (e.g. participant and intervention characteristics, funding sources). Describe any assumptions made about any missing or unclear information.	Page 6-7
Study risk of bias assessment	11	Specify the methods used to assess risk of bias in the included studies, including details of the tool(s) used, how many reviewers assessed each study and whether they worked independently, and if applicable, details of automation tools used in the process.	Page 7, Supple Tab 2
Effect measures	12	Specify for each outcome the effect measure(s) (e.g. risk ratio, mean difference) used in the synthesis or presentation of results.	Page 7
Synthesis methods	13a	Describe the processes used to decide which studies were eligible for each synthesis (e.g. tabulating the study intervention characteristics and comparing against the planned groups for each synthesis (item #5)).	Page 7
	13b	Describe any methods required to prepare the data for presentation or synthesis, such as handling of missing summary statistics, or data conversions.	Page 7
	13c	Describe any methods used to tabulate or visually display results of individual studies and syntheses.	Page 7
	13d	Describe any methods used to synthesize results and provide a rationale for the choice(s). If meta-analysis was performed, describe the model(s), method(s) to identify the presence and extent of statistical heterogeneity, and software package(s) used.	/
	13e	Describe any methods used to explore possible causes of heterogeneity among study results (e.g. subgroup analysis, meta-regression).	/
	13f	Describe any sensitivity analyses conducted to assess robustness of the synthesized results.	/

Section and Topic	Item #	Checklist item	Location where item is reported
Reporting bias assessment	14	Describe any methods used to assess risk of bias due to missing results in a synthesis (arising from reporting biases).	Page 7
Certainty assessment	15	Describe any methods used to assess certainty (or confidence) in the body of evidence for an outcome.	Page 7
**RESULTS**			
Study selection	16a	Describe the results of the search and selection process, from the number of records identified in the search to the number of studies included in the review, ideally using a flow diagram.	Page 7, Fig1
	16b	Cite studies that might appear to meet the inclusion criteria, but which were excluded, and explain why they were excluded.	/
Study characteristics	17	Cite each included study and present its characteristics.	Page 8-9, Tab 1-3, Fig 2, Supple Tab 3-4
Risk of bias in studies	18	Present assessments of risk of bias for each included study.	Page 9-10 Fig 3
Results of individual studies	19	For all outcomes, present, for each study: (a) summary statistics for each group (where appropriate) and (b) an effect estimate and its precision (e.g. confidence/credible interval), ideally using structured tables or plots.	Page 8-9, Tab 1-3, Fig 2, Suppl Tab 5
Results of	20a	For each synthesis, briefly summarise the characteristics and risk of bias among contributing studies.	Page 10-11

Section and Topic	Item #	Checklist item	Location where item is reported
syntheses	20b	Present results of all statistical syntheses conducted. If meta-analysis was done, present for each the summary estimate and its precision (e.g. confidence/credible interval) and measures of statistical heterogeneity. If comparing groups, describe the direction of the effect.	Page 10-11, Fig 4, Suppl Tab 5
	20c	Present results of all investigations of possible causes of heterogeneity among study results.	/
	20d	Present results of all sensitivity analyses conducted to assess the robustness of the synthesized results.	/
Reporting biases	21	Present assessments of risk of bias due to missing results (arising from reporting biases) for each synthesis assessed.	/
Certainty of evidence	22	Present assessments of certainty (or confidence) in the body of evidence for each outcome assessed.	Page 10-11, Fig 4, Suppl Tab 5
**DISCUSSION**			
Discussion	23a	Provide a general interpretation of the results in the context of other evidence.	Page 11-14
	23b	Discuss any limitations of the evidence included in the review.	Page 14-15
	23c	Discuss any limitations of the review processes used.	Page 14-15
	23d	Discuss implications of the results for practice, policy, and future research.	Page 14-15
**OTHER INFORMATION**			
Registration and protocol	24a	Provide registration information for the review, including register name and registration number, or state that the review was not registered.	Page 4

Section and Topic	Item #	Checklist item	Location where item is reported
	24b	Indicate where the review protocol can be accessed, or state that a protocol was not prepared.	/
	24c	Describe and explain any amendments to information provided at registration or in the protocol.	/
Support	25	Describe sources of financial or non-financial support for the review, and the role of the funders or sponsors in the review.	Title page
Competing interests	26	Declare any competing interests of review authors.	Title page
Availability of data, code and other materials	27	Report which of the following are publicly available and where they can be found: template data collection forms; data extracted from included studies; data used for all analyses; analytic code; any other materials used in the review.	Title page

From: Page MJ, McKenzie JE, Bossuyt PM, Boutron I, Hoffmann TC, Mulrow CD, et al. The PRISMA 2020 statement: an updated guideline for reporting systematic reviews. BMJ 2021;372:n71. doi: 10.1136/bmj.n71

For more information, visit: http://www.prisma-statement.org/

© 2024 Lu C.L. et al.
